# *De Novo* modeling of Envelope 2 protein of HCV isolated from Pakistani patient and epitopes prediction for vaccine development

**DOI:** 10.1186/1479-5876-12-115

**Published:** 2014-05-07

**Authors:** Samia Afzal, Muhammad Idrees, Mazhar Hussain

**Affiliations:** 1 Division of Molecular Virology, National Centre of Excellence in Molecular Biology, University of the Punjab, 87-West Canal Bank Road Thokar Niaz Baig, Lahore 53700, Pakistan

**Keywords:** HCV, E2 protein, Epitopes, Modeling

## Abstract

Hepatitis C virus (HCV) is a universal health issue and a significant risk factor leading to hepatocellular carcinoma. HCV has infected approximately 170 million individuals worldwide. It is a member of Flaviviridae with positive sense RNA genome. In the absence of any effective vaccine against HCV, pegylated interferon with ribavirin is the standard of treatment against HCV infection. In this study, sequence and structural analysis of envelope 2 (E2) protein was performed which was isolated from patients of HCV genotype 3a in Pakistan. Then, epitopes were predicted which were specific for both B-cells and T-cells. Later, conservancy of epitopes was checked with the HCV 3a and 1a sequences from different countries. A total of 6 conserved epitopes were found from extra-membranous regions of E2 protein. Presence of conserved epitopes in E2 protein generates the possibility that these epitopes can be used to elicit the immune response against HCV.

## Introduction

Hepatitis C virus (HCV) is a universal health issue and a significant risk factor developing hepatocellular carcinoma (HCC). World Health Organization reported that hepatic cancer caused by HCV scored about 300,000 deaths in 2004 only [1]. It has affected approximately 170 million individuals worldwide [2]. In United States, HCV is most common blood-borne infection with over 4 million individuals infected [3]. According to a recent study, an alarming 17 million people in Pakistan are infected with HCV and about 8-10% of individuals are carriers of HCV pathogen [4]. Prevalence analysis of Pakistani population shows that HCV genotype 3a is the most common genotype in all provinces except in Balochistan where HCV genotype 1a is most prevalent [5].

HCV is a member of Flaviviridae and closely related to Dengue, West Nile and Yellow Fever virus. It has a positive sense single stranded RNA genome of about 9.6 kb size [6]. HCV genome encodes a large polyprotein of 3010 to 3033 amino acid residues [7,8]. This polyprotein is subsequently cleaved into four structural (Core, E1, E2 and P7) and 6 non structural proteins (NS2, NS3, NS4A, NS4B, NS5A and NS5B) [9].

However, the structural details of the HCV virus are still elusive [10], but it is known that infectious viral particles contains lipid envelope and glycoproteins E1 and E2, close to the surface [11]. E2 is highly glycosylated with most of the glycosylation sites well conserved [12]. In addition to these conserved residues, E2 has hypervariable regions which vary up to 80% among HCV of different genotypes and even between the subtypes of same genotype [13]. However, E2 protein interacts with DC-SIGN and L-SIGN (mannose binding proteins) but detailed mechanism of viral entry is unclear. It is suggested that glycosylated motifs of E2 protein interacts with surface receptor enabling the viral entry into the cell [14]. Hence, E2 protein is a potent target to stop viral entry into healthy cells [15].

Currently, the pegylated interferon (IFN) alpha, separately or in combination with ribavirin, is standard HCV treatment [16-18]. The efficacy of IFN treatment depends on many factors related to viral genotype and patient’s health status. Clinical studies show that in 30-50% of cases HCV remain non-responsive to IFN treatment and there might be a number of serious adverse events associated with treatment [9]. In spite of advancements in drug designing technologies, there is still no vaccine against HCV. Variability across the HCV genotypes is also a significant hindrance in vaccine development.

For the development of potential inhibitors against envelope proteins, it is required to have knowledge of sequence and structure of protein. With the development of computational biology, novel approaches have been developed to get insights from biological data. This study was designed to isolate E2 glycoprotein sequence from HCV genotype 3a infected patient; and to predict and to analyze the epitopes related to B-cells and T-cells. The conservation and variability analysis was included to find spectrum of activity of epitopes in HCV genotype 3a and 1a.

## Methodology

### Source of serum samples

HCV genotype 3a samples were collected from patients diagnosed with HCV at Molecular Diagnostics Lab. CEMB, Lahore. The informed consent was taken from the patients and blood sample was taken according to provision of Ethical Committee, Molecular Virology Division, National Centre of Excellence in Molecular Biology, Lahore. The patients were selected on the basis of elevated serum ALT and AST levels at least for six months, histological examination, and detection of serum HCV RNA anti-HCV antibodies (3rd generation ELISA).

### RNA isolation from serum, cDNA synthesis and sequencing of HCV E2 gene

Total RNA was isolated from patients’ serum samples using Gentra RNA isolation kit (Puregene, Minneapolis, MN55441 USA) according to the kit protocol. Then, extracted RNA was reverse transcribed to cDNA using MMLV-RTase (Moloney murine leukemia virus reverse transcriptase). By using the E2 specific primers, E2 gene was amplified using cDNA of HCV genotype 3a. For this, PCR protocol involved 35 cycling steps at annealing temperature 54°C. The amplified PCR product was resolved using 1.2% TAE agarose gel and molecular weight was compared with 1 kb DNA ladder. Then, DNA was purified from gel using QIA quick gel extraction kit (Qiagen, USA) using the kit protocol. Purified PCR product was cloned in pCR2.1-TOPO (TA vectors) obtained from Invitrogen, USA. Successful cloning was confirmed by PCR using E2 specific primers and by digestion of construct using EcoR1 at 37°C for 1 hr. Later, a sequencing reaction was performed using BigDye™ Terminator v3.0 sequencing kit (Applied Biosystems, Germany). Both positive and negative strands were sequenced at automated sequencer (Applied Biosystems 3700 DNA Analyzer, Germany). Then, the gene sequence was submitted at NCBI GenBank, having accession no. ADP55199.

### Sequence analysis of E2 protein

HCV 3a E2 gene sequence ADP55199 was used for primary structure analysis and for the prediction of secondary as well as three-dimensional structure. The E2 gene sequence was in-*silico* translated to obtain primary structure (amino acid sequence) of protein. Primary structure parameters of E2 protein which include molecular weight, theoretical pI, atomic composition, extinction coefficient, estimated half-life, aliphatic index and Grand average of hydropathicity (GRAVY) were computed using ProtParam online tool [19]. Secondary structure of the protein was analyzed using Jpred, Psipred and “Sequence Annotated by Structure” (SAS) tool [20-22]. Disulfide connectivity of the protein was checked using DiANNA tool which is a neural network application and predicts cysteine states of a protein [23]. The knowledge of cys-cys linkages is important in understanding the secondary and tertiary structure of protein because it plays significant role in fold stabilization. Glycosylation sites were predicted using NetNGlyc 1.0 server and their conservancy was checked using multiple sequence alignment by MEGA5.0 [24].

### *De novo* protein modeling and quality assessment

For the prediction of three dimensional structure of E2 protein both homology modeling and *de novo* modeling approaches were used. For the homology modeling, BlastP was used for searching suitable template in Protein Data Bank (http://www.rcsb.org/pdb/home/home.do). In our search, the appropriate template was not found, so we used iTASSER server for *de novo* modeling of E2 protein [25]. Using iTASSER, five models were predicted and one best model was chosen for further structural analysis. The selection of model was done using three criteria: C-score, DFIRE2 energy profile [26] and stereochemical properties using PROCHECK tool [27]. The visual analysis of structure was done on Swiss-PDB-viewer [28] and Visual Molecular Dynamics (VMD) program [29].

### Epitopes prediction from E2 protein

A systematic approach was employed for the prediction of potential epitopes in E2 protein. Vexijen 1.0 was used to determine overall antigenicity of E2 protein using cut-off value of 0.4 [30]. Then, topology of E2 protein was determined using TMHMM Server v 2.0 [31]. With the help of membrane topology data, E2 protein regions inside the membrane and transmembrane were eradicated from epitopes prediction. BCPRED server was used for the prediction of B-cell epitopes of the length of 12 amino acids [32]. For the prediction of T-cell epitopes ProPred was used with proteasome cleavage site filter of 5% threshold. In this analysis, 47 alleles of MHC-class I and 54 alleles of MHC-class II were included [33]. Once the B-cells and T-cells (MHC-class I and MHC-class II) epitopes were predicted, their antigenicity was checked using Vexijen. The antigenicity score of the predicted epitopes was checked using Vexijen v 1.0 server. Later, only antigenic epitopes were included in conservancy analysis.

### The conservancy of epitopes

The E2 protein sequences of HCV genotype 3a and 1a were retrieved from NCBI sequence database. The HCV 3a sequences were from India (AGQ17416), Japan (BAN67274), United Kingdom (ACZ61116) and USA (ABD85062) and HCV 1a sequences were from Pakistan (GU736411), USA (EU482831), United Kingdom (AY958057), France (AF529293) and Japan (AB520610). The conservancy and variability of the predicted antigenic epitopes was determined by “IEDB conservancy analysis tool” [34] in E2 protein sequences retrieved from different regions of world. Then, all highly conserved epitopes were checked for their localization in predicted protein structure.

## Results and discussion

### cDNA synthesis and cloning of E2 protein

**Figure 1:**
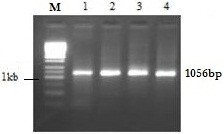
**PCR amplification of HCV E2 protein from HCV patients.** The gel shows 1056 bp (1 – 4) gene products and their molecular weight is compared to 1 kb ladder (M).

**Figure 2:**
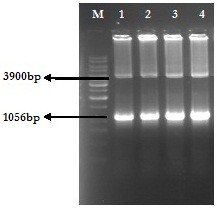
**EcoR1 digestion of E2 carrying pCR2.1 vectors.** For the comparison of DNA sizes, 1 kb ladder (M) is used. The restriction digestion reactions (1 – 4) shows 1056 bp band of E2 gene and 3900 bp band of linearized pCR2.1 (TA) vector.

Serum samples collected from HCV genotype 3a positive individuals were processed for total RNA extraction. Complimentary DNA was prepared from RNA and then E2 gene was amplified using gene specific primers. The PCR product was checked on the gel and bands were observed at 1056 bp position with reference to 1 kb DNA marker (Figure 1). DNA in the gel was used for ligation in pCR2.1 for TA cloning. TA cloning was verified by PCR using E2 gene specific primers and *Eco*R1 digestion. The restriction digestion with EcoR1 produced 2 bands for each sample (Figure 2). A band with 3.9 kb molecular weight indicates the linearized pCR2.1 and the other with ~1.05 kb indicates the size of E2 gene. One sample was further processed for sequencing, structural and epitope prediction.

### Sequence analysis of E2 protein

**Figure 3:**
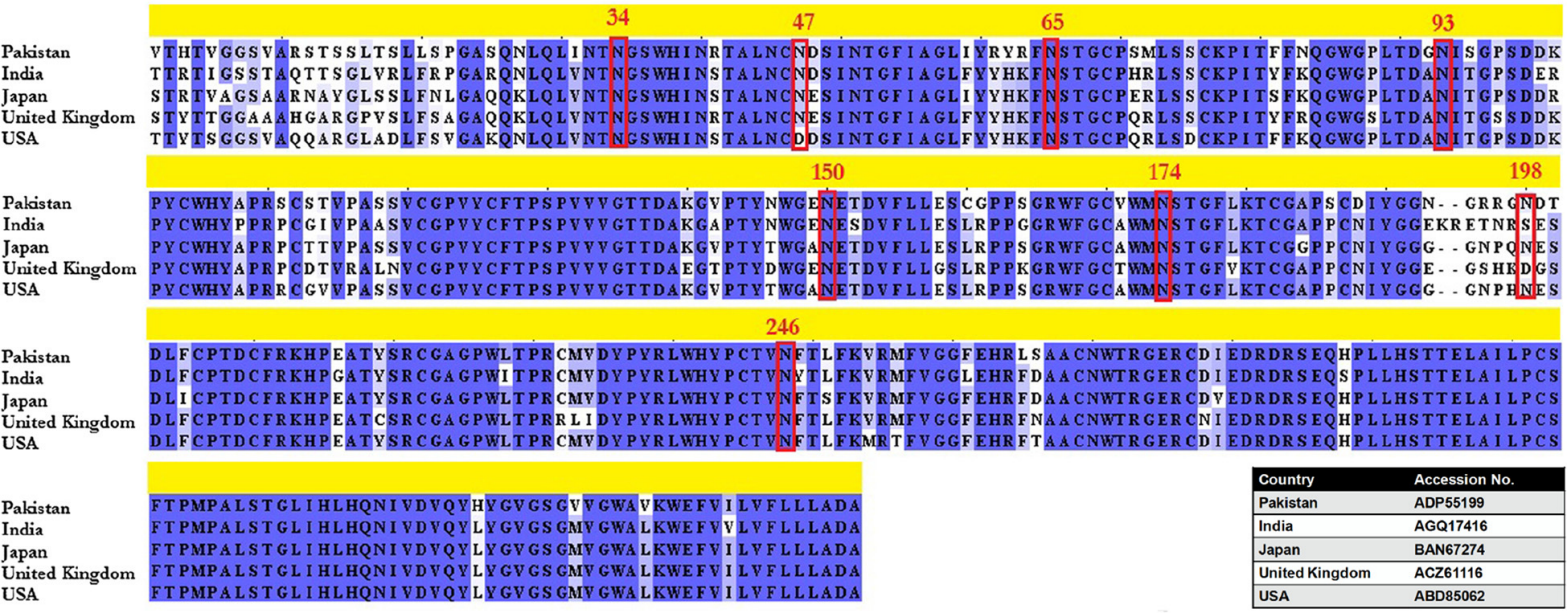
The glycosylation sites prediction and conservancy in these sites in E2 proteins from different regions of world.

In this study, we have employed sequence analysis, de novo modeling and epitopes prediction from E2 protein isolated from Pakistani patient. Its estimated molecular weight is 38703.8 daltons and comprised of 352 amino acids. The pI and extinction coefficient of the protein were 6.86 and 80995 M^-1^ cm^-1^, respectively. The protein has GRAVY score of -0.122 which indicates that protein is hydrophilic in nature. Glycosylation sites analysis revealed the presence of eight potential glycosylation sites in the protein (Figure 3). Among all, six sites at positions 34, 65, 93, 150, 174 and 246 were conserved in the sequences retrieved from various regions of the world. The protein also contains 9 disulfide bridges (Table 1) which renders extracellular stability in proteins [35,36].

**Table 1:** Predicted disulfide bonds

**Predicted bonds**	
46 – 267	RTALNCNDSIN – RLSAACNWTRG
69 – 104	FNSTGCPSMLS – DDKPYCWHYAP
76 – 187	SMLSSCKPITF – CGAPSCDIYGG
112 – 275	YAPRSCSTVPA – TRGERCDIEDR
121 – 170	PASSVCGPVYC – GRWFGCVWMNS
126 – 208	CGPVYCFTPSP – FCPTDCFRKHP
160 – 204	FLLESCGPPSG – DTDLFCPTDCF
220 – 300	ATYSRCGAGPW – LAILPCSFTPM
230 – 243	WLTPRCMVDYP – LWHYPCTVNFT

### *De novo* models and quality assessment

**Figure 4:**
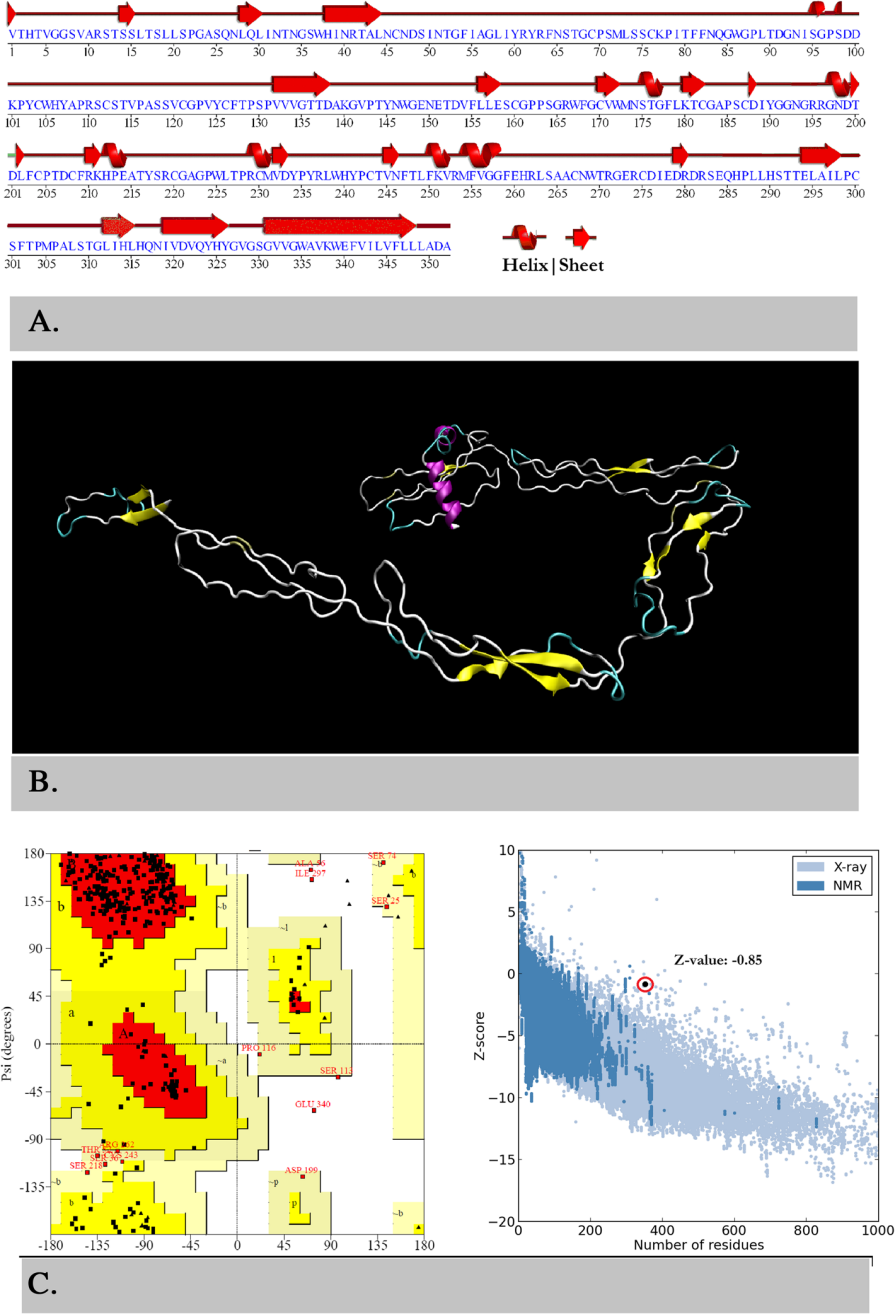
**Sequence and structural analysis of E2 protein. (A)** Secondary structure of the protein with respect to protein sequence and **(B)** Ribbon representation of 3D structure of E2 protein selected model. Ramachandran plot **(C)** is showing the phi and psi angles distribution in the protein while z-score **(D)** is representing the overall quality of model with respect to high resolution models.

Secondary structure of the protein shows that loops make the 78% of the protein while sheets and helices contribute 14% and 8% of the structure, respectively (Figure 4A). Using iTASSER server, five protein models were developed. The best model was selected by combined evaluation based on C-score (confidence score from iTASSER server), DFIRE2 energy profile and stereochemical properties using Ramachandran plot. It is very evident from Table 2 that Model1 is best among all others based on C-score and Ramachandran plot. In addition to this, secondary structures in three-dimensional structure of Model1 have stronger correlation with predicted secondary structures with other methods (Figure 4B). As far as, Model1 stereochemical quality is concerned, it showed 82.1% amino acid residues in allowed and only 1.4% residues in disallowed region of Ramachandran plot (Figure 4C). However, its DFIRE2 score was not better than other 4 models but its z-score (-0.85) based on its overall energy was comparable with non-redundant set of high quality models (Figure 4D). On these bases, model1 was chosen for the structural analysis of E2 protein.

**Table 2:** Assessment of iTASSER predicted E2 protein models

	**Model 1**	**Model 2**	**Model 3**	**Model 4**	**Model 5**
I-TASSER (C-score)	-1.17	-3.11	-3.12	-3.17	-3.35
DFIRE2	-420.54	-586.18	-573.31	-579.41	-599.08
PROCHECK					
Allowed	82.1%	69.4%	72.9%	69.8%	70.4%
Additionally allowed	13.7%	19.6%	17.9%	19.6%	20.3%
Generously allowed	2.7%	4.1%	5.2%	5.5%	3.8%
Disallowed	1.4%	6.9%	4.1%	5.2%	5.5%

### Epitopes prediction for the vaccine development

Humoral and cellular immunity are the two arms of immunity provided by B-cells and T-cells, respectively. The recognition of pathogenic epitopes by B-cell and T-cells lies at the heart of immune response. Such recognition starts the mechanism of activation of humoral and cellular response and leads to ultimate destruction of pathogenic organism [35]. Epitopes are principal components of both subunit and poly-epitopic vaccines. Thus, it is a pivotal challenge for immune-informatics to accurately predict the B-cell and T-cells epitopes [37].

It is important for a protein to expose in outside environment to interact with soluble antibodies, B-cells and T-cells. So, the membrane topology of the E2 protein was determined using TMHMM server. In this analysis, a total of 2 transmembrane helices with, 23 amino acids each, were predicted. First helix spans from residue 293 to residue 315 while second helix spans from residue 328 to residue 350. A large 1 to 292 portion of the protein was outside of the membrane and a small loop like structure was present inside the membrane. For the prediction of B-cell and T-cell epitopes, only extra-membranous region was selected. The antigenicity score of selected (1 – 292) region was 0.4911 which indicated that this region as a probable antigen.

#### *B-cell epitopes prediction*

B-cell epitopes are important to elicit humoral immune response. B-Cell epitopes were predicted using BCPred server. Using the configuration of 12 amino acids, 18 epitopes were predicted using extra-membranous region of the protein (Table 3). Then, all epitopes were checked for antigenicity using Vexijen server. Among them 13 were having antigenicity score greater than 0.4 threshold value for antigens while 5 were below this limit. Epitope DIYGGNGRRGND present at position 188 had highest antigenicity score. A higher antigenicity score indicates the better binding affinity between receptor and epitope [36].

**Table 3:** B-cell epitopes with their antigenicity score

**Starting position**	**Epitope**	**BCPreds score**	**Antigenicity score**
160	**CGPPSGRWFGCV**	**0.998**	**-0.7569**
82	FNQGWGPLTDGN	0.969	0.9925
188	DIYGGNGRRGND	0.965	1.4612
136	TTDAKGVPTYNW	0.93	0.4148
29	QLINTNGSWHIN	0.916	0.7087
278	EDRDRSEQHPLL	0.892	0.5071
108	**APRSCSTVPASS**	**0.849**	**-0.0164**
221	GAGPWLTPRCMV	0.827	0.4537
203	**FCPTDCFRKHPE**	**0.819**	**-0.1963**
123	PVYCFTPSPVVV	0.812	0.9481
60	YRYRFNSTGCPS	0.806	0.9192
43	ALNCNDSINTGF	0.679	0.8184
95	SGPSDDKPYCWH	0.645	1.2504
174	**NSTGFLKTCGAP**	**0.587**	**-0.3698**
301	SFTPMPALSTGL	0.56	0.6394
234	YPYRLWHYPCTV	0.383	0.4361
265	AACNWTRGERCD	0.311	0.4919
252	**VRMFVGGFEHRL**	**0.291**	**0.1934**

Non-Antigens are shown in bold face.

#### *T cell epitopes prediction*

T-cell epitopes are presented on either MHC class I or MHC class II which play significant role in cell-mediated immunity [38,39]. For the prediction of T-cell epitopes, we used ProPred server with 47 alleles for MHC class I and 54 alleles for MHC class II. A total of 4 epitopes were predicted for MHC class I in extra-membranous region while only 2 epitopes, FFNQGWGPL and TPSPVVVGT, showed antigenicity score greater than antigenicity threshold (0.4) (Table 4). Similarly, 34 epitopes were predicted for MHC class II and 24 among them were found to be antigenic (Table 5). Out of 24 antigenic peptides, eleven showed antigenicity score >1.000 which is considered good in terms of stability of receptor and epitope interaction.

**Table 4:** MHC Class I epitopes with their antigenicity scores

**Starting position**	**Peptide**	**Allele**	**Antigenicity score**
81	FFNQGWGPL	HLA-A24, HLA-B_3801, HLA-B_3902, HLA-B_5301, HLA-B_5401, HLA-B_51, HLA-B_0702, HLA-Cw_0401, MHC-Kd	1.0281
128	TPSPVVVGT	HLA-B_3501, HLA-B_5101, HLA-B_5102, HLA-B_5103, MHC-Ld	1.5496
12	**STSSLTSLL**	**HLA-B**_**3701, HLA-B**_**3902, HLA-B**_**5801, HLA-B60, HLA-B7, HLA-Cw**_**0602, MHC-Kb**	**0.0685**
1	**VTHTVGGSV**	**HLA-B**_**5103, HLA-B**_**5301, HLA-B**_**51, HLA-B**_**5801, HLA-B61**	**0.2406**

Non-Antigens are shown in bold face.

**Table 5:** MHC Class II epitopes with antigenicity scores

**Starting position**	**Peptide**	**Allele**	**Antigenicity score**
28	**LQLINTNGS**	**DRB1_0101, DRB1_0102, DRB1_0301, DRB1_0305, DRB1_0306, DRB1_0307, DRB1_0308, DRB1_0309, DRB1_0311, DRB1_0401, DRB1_0402, DRB1_0404, DRB1_0405, DRB1_0408, DRB1_0410, DRB1_0421, DRB1_0423, DRB1_0426, DRB1_0802, DRB1_0804, DRB1_0806, DRB1_0813, DRB1_1101, DRB1_1102, DRB1_1104, DRB1_1106, DRB1_1107, DRB1_1114, DRB1_1120, DRB1_1121, DRB1_1128, DRB1_1301, DRB1_1302, DRB1_1304, DRB1_1305, DRB1_1307, DRB1_1311, DRB1_1321, DRB1_1322, DRB1_1323, DRB1_1327, DRB1_1328, DRB1_1506, DRB5_0101, DRB5_0105**	**-0.2636**
64	FNSTGCPSM	DRB1_0101, DRB1_0701, DRB1_0703,	0.6645
179	**LKTCGAPSC**	**DRB1_0101, DRB1_0102,**	**-0.2346**
31	INTNGSWHI	DRB1_0102, DRB1_0402, DRB1_0701, DRB1_0703,	1.0793
124	VYCFTPSPV	DRB1_0102, DRB1_0423, DRB1_1501, DRB1_1506	1.4124
154	**VFLLESCGP**	**DRB1_0102, DRB1_0404, DRB1_0408, DRB1_0410, DRB1_0421, DRB1_0423,**	**-1.3987**
58	**LIYRYRFNS**	**DRB1_0301, DRB1_0305, DRB1_0309, DRB1_0801, DRB1_0802, DRB1_0804, DRB1_0806, DRB1_0813, DRB1_0817, DRB1_1101, DRB1_1102, DRB1_1104, DRB1_1106, DRB1_1107, DRB1_1114, DRB1_1120, DRB1_1121, DRB1_1301, DRB1_1302, DRB1_1304, DRB1_1307, DRB1_1311, DRB1_1322, DRB1_1323, DRB1_1327, DRB1_1328, DRB1_1501, DRB1_1506**	**-0.051**
72	**MLSSCKPIT**	**DRB1_0301, DRB1_0305, DRB1_0309, DRB1_1107**	**-0.3409**
132	VVVGTTDAK	DRB1_0301, DRB1_0305, DRB1_0306, DRB1_0307, DRB1_0308, DRB1_0311, DRB1_1107,	1.8645
37	**WHINRTALN**	**DRB1_0305, DRB1_0309, DRB1_0401, DRB1_0405, DRB1_0408, DRB1_0410, DRB1_0421, DRB1_0426, DRB1_0801, DRB1_0802, DRB1_0806, DRB1_0817, DRB1_1101, DRB1_1114, DRB1_1120, DRB1_1128, DRB1_1302, DRB1_1304, DRB1_1305, DRB1_1307, DRB1_1321, DRB1_1323**	**0.3868**
62	YRFNSTGCP	DRB1_0305, DRB1_0309, DRB1_0401, DRB1_0405, DRB1_0408, DRB1_0421, DRB1_0426, DRB1_0801, DRB1_0802, DRB1_1101, DRB1_1120, DRB1_1128, DRB1_1302, DRB1_1305, DRB1_1307, DRB1_1321,	0.9493
105	WHYAPRSCS	DRB1_0305, DRB1_0306, DRB1_0307, DRB1_0308, DRB1_0309, DRB1_0311, DRB1_0801, DRB1_0802, DRB1_0813, DRB1_1101, DRB1_1107, DRB1_1114, DRB1_1120, DRB1_1128, DRB1_1302, DRB1_1305, DRB1_1307, DRB1_1323	0.5807
172	**WMNSTGFLK**	**DRB1_0305, DRB5_0101, DRB5_0105**	**0.1523**
169	WTRGERCDI	DRB1_0305, DRB1_0309,	0.9138
54	FIAGLIYRY	DRB1_0309,	0.4518
127	FTPSPVVVG	DRB1_0309, DRB1_0421	1.3553
239	WHYPCTVNF	DRB1_0309, DRB1_0405, DRB1_0421, DRB1_0703	0.7657
167	**WFGCVWMNS**	**DRB1_0401, DRB1_0408, DRB1_1101**	**0.2989**
60	YRYRFNSTG	DRB1_0402, DRB1_0405, DRB1_0408, DRB1_0421, DRB1_0801, DRB1_0802, DRB1_0806, DRB1_0813, DRB1_0817, DRB1_1120, DRB1_1302, DRB1_1502	1.5053
247	FTLFKVRMF	DRB1_0402, DRB1_0701, DRB1_0703, DRB1_0801, DRB1_0802, DRB1_1101, DRB1_1102, DRB1_1114, DRB1_1120, DRB1_1121, DRB1_1128, DRB1_1301, DRB1_1302, DRB1_1304, DRB1_1305, DRB1_1307, DRB1_1323, DRB1_1327, DRB1_1328	0.0943
256	VGGFEHRLS	DRB1_0402, DRB1_1102, DRB1_1114, DRB1_1120, DRB1_1121, DRB1_1301, DRB1_1302, DRB1_1304, DRB1_1322, DRB1_1323, DRB1_1327, DRB1_1328	1.3166
252	**VRMFVGGFE**	**DRB1_0405, DRB1_0410, DRB1_0801, DRB1_0806, DRB1_0817, DRB1_1304, DRB1_1321, DRB1_1501, DRB1_1502, DRB1_1506**	**-0.4355**
147	**WGENETDVF**	**DRB1_0421**	**-0.0038**
125	YCFTPSPVV	DRB1_0701, DRB1_0703	1.2112
241	YPCTVNFTL	DRB1_0701, DRB1_0703	0.9270
259	FEHRLSAAC	DRB1_0801, DRB1_0802, DRB1_0813	1.8179
277	IEDRDRSEQ	DRB1_0813	0.5283
103	YCWHYAPRS	DRB1_1114, DRB1_1120, DRB1_1302, DRB1_1323	1.3992
190	YGGNGRRGN	DRB1_1114, DRB1_1120, DRB1_1302, DRB1_1304, DRB1_1323	2.2420
209	FRKHPEATY	DRB1_1114, DRB1_1120, DRB1_1302, DRB1_1323,	0.8056
236	YRLWHYPCT	DRB1_1502, DRB1_1506	0.5131
245	VNFTLFKVR	DRB5_0101, DRB5_0105	0.9765
59	IYRYRFNST	DRB1_1501, DRB1_1502, DRB1_1506	0.9405
55	IAGLIYRYR	DRB5_0101, DRB5_0105	1.4380

Non-Antigens are shown in bold face.

### Conservancy and structural analysis of predicted epitopes

**Figure 5:**
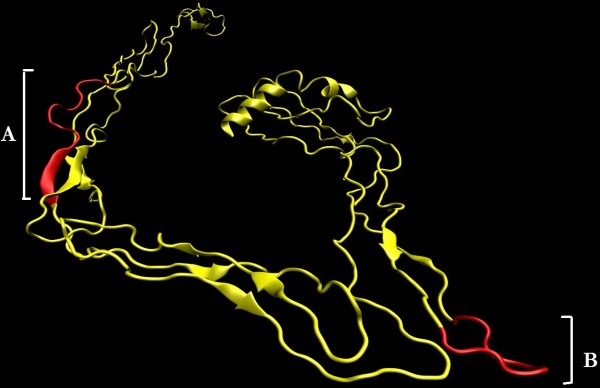
**Structural analysis of highly conserved epitopes in HCV E2 protein.** The structure revealed that it contains overlapping epitopes **(A)** region from 123 – 136 related to B-cells and T-cells. On the other hand, a single epitope is also present at position 236 **(B)** which is related to T-cell.

The assessment of epitope conservation is important in vaccine design because a higher level of conservation ensure broader protection against multiple strains of a pathogen [34]. The conservancy of the predicted epitopes was determined using E2 proteins from HCV 3a and HCV 1a from various regions of the world. For this, 3a sequence from India, Japan, United Kingdom and USA; and 1a sequence from Pakistan, France, Japan, UK and USA were downloaded from NCBI database. The IEDB conservancy analysis was used to determine conserved epitopes among all selected E2 sequences from genotypes 3a and 1a (Additional file 1: Table S1). Separate analysis of HCV genotype 3a and 1a epitopes showed conservancy in 9 epitope sequences each. Further analysis showed that six epitopes were highly conserved for both HCV genotype 3a and 1a (Table 6). Among 6 conserved epitopes, a B-cell epitope and an MHC-class I (T-cell) epitope was conserved at positions 123 and 128, respectively. Furthermore, four MHC-class II (T-cell) epitopes were found conserved at positions 124, 125, 127 and 236. This indicates that E2 protein can elicit not only humoral immune response but also helper-T cells and cytotoxic-T cells too. The structural analysis of E2 protein showed that all epitopes were exposed to the surface (Figure 5). Surface exposure of epitopes is important for interaction with respective immune cell receptors [35,36,40].

**Table 6:** Highly conserved epitopes from E2 of 3a and 1a

**Epitopes**	**B-cell/T-cell**
PVYCFTPSPVVV	B-cell
TPSPVVVGT	T-cell (HLA-B_3501, HLA-B_5101, HLA-B_5102, HLA-B_5103, MHC-Ld)
VYCFTPSPV	T-cell (DRB1_0102, DRB1_0423, DRB1_1501, DRB1_1506)
FTPSPVVVG	T-cell (DRB1_0309, DRB1_0421)
YCFTPSPVV	T-cell (DRB1_0701, DRB1_0703)
YRLWHYPCT	T-cell (DRB1_1502, DRB1_1506)

Moreover, it is generally desirable that a vaccine formulation may have one or more B-cell and T-cell epitopes because a wide immune response can efficiently eradicate the invading pathogen. Sometimes, a small protein motif with overlapping epitopes for B-cells and T-cells can stimulate the humoral and cell-mediated immune response. The conserved epitopes in HCV E2 protein showed that the loop-sheet motif from 123 to 136 region contains 5 overlapping epitopes for both B-cells and T-cells (Figure 5). This motif has one B-cell and 4 T-cell epitopes. T-cell epitopes include one MHC class I specific and three MHC class II specific epitopes. Hence, this motif of 13 amino acids can induce broad immune response against HCV pathogen.

## Conclusion

HCV is prevalent worldwide and there is no vaccine developed against this virus. There are multiple antigenic components which can be used for vaccine development. In Pakistan, HCV genotype 3a is most common followed by 1a. The sequence, structural and epitope analysis has revealed a number of conserved epitopes in both 3a and 1a genotypes. These epitopes may not only help in diagnosing the pathogens but also may help in developing vaccine against HCV 3a and 1a. Presence of overlapping epitopes generates the hope that a small fragment of peptide in vaccine formulation can elicit broad immune response and may result in efficient clearing of pathogen.

## Competing interests

The authors declare that they have no competing interests.

## Authors’ contributions

MI conceived of the study. SA performed the experimental work, organised the data and drafted the manuscript. MH performed the evolutionary and statistical analysis. All the authors read and approved the final manuscript.

## Supplementary Material

Additional file 1: Table S1 Conservancy of E2 protein epitopes with HCV 3a and HCV 1a sequences from various countries. Click here for file
