# Supplementary material for: De Novo modeling of Envelope 2 protein of HCV isolated from Pakistani patient and epitopes prediction for vaccine development
Source: J Transl Med. 2014 May 7;12:115. doi: 10.1186/1479-5876-12-115 (PMC4024208; doi:10.1186/1479-5876-12-115)
Supplement: Additional file 1: Table S1 — Conservancy of E2 protein epitopes with HCV 3a and HCV 1a sequences from various countries. [file 1479-5876-12-115-S1.docx]

**Supplementary Table 1. Conservancy of E2 protein epitopes with HCV 3a and HCV 1a sequences from various countries**

| Peptide | 3a genotype | | | | | Minimum Identity | 1a genotype | | | | | Minimum Identity |
| --- | --- | --- | --- | --- | --- | --- | --- | --- | --- | --- | --- | --- |
|  | **India** | **Japan** | **United Kingdom** | | **United States** |  | **Pakistan** | **United States** | **United Kingdom** | **France** | **Japan** |  |
| YGVGSGVVGWAV | YGVGSG**M**VGWA**L** | YGVGSG**M**VGWA**L** | YGVGSG**M**VGWA**L** | YGVGSG**M**VGWA**L** | | 83.33% | YGVGS**S**VV**S**WA**I** | YGVGS**SI**V**S**WA**I** | YGVGS**SI**V**S**WA**I** | YGVGS**SI**V**S**WA**I** | YGVGS**SI**V**S**WA**I** | 66.67% |
| FNQGWGPLTDGN | F**K**QGWGPLTD**A**N | F**K**QGWGPLTD**A**N | F**R**QGWG**S**LTD**A**N | F**K**QGWGPLTD**A**N | | 75.00% | F**D**QGWGP**ISYT**N | F**D**QGWGP**ISYA**N | F**D**QGWGP**I**T**HA**N | F**A**QGWGP**ISHV**N | F**D**QGWGP**IRHA**N | 58.33% |
| DIYGGNGRRGND | **N**IYGG**EK**R**ET**N**R** | **N**IYGG**G**G**NPQ**N**E** | **N**IYGG**E**G**SHKDG** | **N**IYGG**G**G**NPH**N**E** | | 41.67% | **CVI**GG**V**G**NNTLH** | **CVI**GG**A**G**NNTLH** | **CVI**GG**V**G**NNTLH** | **GGV**G**N**N**TL**R**CPT** | Absent | 0.00% |
| TTDAKGVPTYNW | TTDAKG**A**PTYNW | TTDAKGVPTY**T**W | TTDA**E**G**T**PTY**D**W | TTDAKGVPTY**T**W | | 75.00% | TTD**RA**GVPTYNW | TTD**KA**G**A**PTYNW | TTD**VL**GVPTY**T**W | TTD**KL**G**A**PTYNW | TT**NRL**GVPTY**S**W | 66.67% |
| QLINTNGSWHIN | QL**V**NTNGSWHIN | QL**V**NTNGSWHIN | QL**V**NTNGSWHIN | QL**V**NTNGSWHIN | | 91.67% | QLINTNGSWHIN | QLINTNGSWHIN | QLINTNGSWHIN | QLINTNGSWHIN | QLINTNGSWHIN | 100.00% |
| EDRDRSEQHPLL | EDRDRSEQ**S**PLL | EDRDRSEQHPLL | EDRDRSEQHPLL | EDRDRSEQHPLL | | 91.67% | **D**DRDRSE**LS**PLL | **D**DRDRSE**LS**PLL | **D**DRDRSE**LS**PLL | **D**DRDRSE**LS**PLL | **D**DRDRSE**LS**PLL | 75.00% |
| GAGPWLTPRCMV | GAGPW**I**TPRCMV | GAGPWLTPRCMV | GAGPWLTPR**RLI** | GAGPWLTPRCMV | | 75.00% | G**S**GPW**I**TPRC**L**V | G**S**GPW**I**TPRC**L**V | G**S**GPW**I**TPRC**L**V | G**S**GPW**I**TPRC**L**V | G**S**GPW**I**TPRC**L**V | 75.00% |
| PVYCFTPSPVVV | PVYCFTPSPVVV | PVYCFTPSPVVV | PVYCFTPSPVVV | PVYCFTPSPVVV | | 100.00% | PVYCFTPSPVVV | PVYCFTPSPVVV | PVYCFTPSPVVV | PVYCFTPSPVVV | PVYCFTPSPVVV | 100.00% |
| YRYRFNSTGCPS | Y**YHK**FNSTGCP**H** | Y**YHK**FNSTGCP**E** | Y**YHK**FNSTGCP**Q** | Y**YHK**FNSTGCP**Q** | | 66.67% | Y**YHK**FNS**S**GCP**E** | Y**HH**RFNSSGCP**E** | Y**YHK**FNS**S**GCP**E** | Y**YNK**FNS**S**GCP**E** | Y**YHK**FNS**S**GCP**E** | 58.33% |
| ALNCNDSINTGF | ALNCNDSINTGF | ALNCN**E**SINTGF | ALNCN**E**SINTGF | ALNC**D**DSINTGF | | 91.67% | **ALNCNGSLDA**G**W** | ALNCN**A**S**LD**TG**W** | ALNCN**A**S**LD**TG**W** | ALNCN**A**S**LE**TG**W** | ALNCN**A**S**LD**TG**W** | 58.33% |
| SGPSDDKPYCWH | **T**GPSD**ER**PYCWH | **T**GPSDD**R**PYCWH | **T**G**S**SDDKPYCWH | **T**GPSDDKPYCWH | | 75.00% | **GSGPEHR**PYCWH | **GSGPEHR**PYCWH | **GSGPEHR**PYCWH | **GSGPEQR**PYCWH | **GSGPEHR**PYCWH | 41.67% |
| SFTPMPALSTGL | SFTPMPALSTGL | SFTPMPALSTGL | SFTPMPALSTGL | SFTPMPALSTGL | | 100.00% | SFT**TL**PAL**T**TGL | SFT**TL**PAL**T**TGL | SFT**TL**PAL**T**TGL | SFT**TL**PAL**T**TGL | SFT**TL**PAL**T**TGL | 75.00% |
| YPYRLWHYPCTV | YPYRLWHYPCTV | YPYRLWHYPCTV | YPYRLWHYPCTV | YPYRLWHYPCTV | | 100.00% | YPYRLWHYPCT**I** | Y**A**YRLWHYPCTV | YPYRLWHYPCTV | YPYRLWHYPCTV | YPYRLWHYPCTV | 91.67% |
| AACNWTRGERCD | AACNWTRGERCD | AACNWTRGERCD | AACNWTRGERC**N** | AACNWTRGERCD | | 91.67% | AACNWTRGERCD | **V**ACNWTRGERCD | AACNWTRGERCD | AACNWTRGERCD | **V**ACNWTRGERCD | 91.67% |
| FFNQGWGPL | **Y**F**K**QGWGPL | **S**F**K**QGWGPL | **Y**F**R**QGWG**S**L | FF**K**QGWGPL | | 66.67% | **D**F**D**QGWGP**I** | **D**F**D**QGWGP**I** | **D**F**D**QGWGP**I** | **Y**F**A**QGWGP**I** | **D**F**D**QGWGP**I** | 66.67% |
| TPSPVVVGT | TPSPVVVGT | TPSPVVVGT | TPSPVVVGT | TPSPVVVGT | | 100.00% | TPSPVVVGT | TPSPVVVGT | TPSPVVVGT | TPSPVVVGT | TPSPVVVGT | 100.00% |
| FNSTGCPSM | FNSTGCP**HR** | FNSTGCP**ER** | FNSTGCP**QR** | FNSTGCP**QR** | | 77.78% | FNS**S**GCP**ER** | FNS**S**GCP**ER** | FNS**S**GCP**ER** | FNS**S**GCP**ER** | FNS**S**GCP**ER** | 66.67% |
| INTNGSWHI | **V**NTNGSWHI | **V**NTNGSWHI | **V**NTNGSWHI | **V**NTNGSWHI | | 88.89% | INTNGSWHI | INTNGSWHI | INTNGSWHI | INTNGSWHI | INTNGSWHI | 100.00% |
| VYCFTPSPV | VYCFTPSPV | VYCFTPSPV | VYCFTPSPV | VYCFTPSPV | | 100.00% | VYCFTPSPV | VYCFTPSPV | VYCFTPSPV | VYCFTPSPV | VYCFTPSPV | 100.00% |
| VVVGTTDAK | VVVGTTDAK | VVVGTTDAK | VVVGTTDA**E** | VVVGTTDAK | | 88.89% | VVVGTTD**RA** | VVVGTTD**KA** | VVVGTTD**VL** | VVVGTTD**KL** | VVVGTT**NRL** | 66.67% |
| YRFNSTGCP | **HK**FNSTGCP | **HK**FNSTGCP | **HK**FNSTGCP | **HK**FNSTGCP | | 77.78% | **HK**FNS**S**GCP | **H**RFNS**S**GCP | **HK**FNS**S**GCP | **NK**FNS**S**GCP | **HK**FNS**S**GCP | 66.67% |
| WHYAPRSCS | WHY**P**PR**P**C**G** | WHYAPR**P**C**T** | WHYAPR**P**C**D** | WHYAPR**R**C**G** | | 66.67% | WHY**P**P**KP**C**G** | WHY**P**P**KP**C**G** | WHY**P**P**KP**C**G** | WHYAPR**P**C**G** | WHY**P**P**KP**C**G** | 55.56% |
| WTRGERCDI | WTRGERCDI | WTRGERCDV | WTRGERCNI | WTRGERCDI | | 88.89% | WTRGERCD**L** | WTRGERCD**L** | WTRGERCD**L** | WTRGERCD**L** | WTRGERCD**L** | 88.89% |
| FIAGLIYRY | FIAGL**F**Y**YH** | FIAGLIY**YH** | FIAGL**F**Y**YH** | FIAGL**F**Y**YH** | | 66.67% | **W**I**V**GL**F**Y**YH** | **W**IAGL**F**Y**HH** | **WV**AGL**F**Y**YH** | **W**IAGL**F**Y**YN** | **WV**AGL**F**Y**YH** | 44.44% |
| FTPSPVVVG | FTPSPVVVG | FTPSPVVVG | FTPSPVVVG | FTPSPVVVG | | 100.00% | FTPSPVVVG | FTPSPVVVG | FTPSPVVVG | FTPSPVVVG | FTPSPVVVG | 100.00% |
| WHYPCTVNF | WHYPCTVN**Y** | WHYPCTVNF | WHYPCTVNF | WHYPCTVNF | | 88.89% | WHYPCT**I**N**Y** | WHYPCTVN**Y** | WHYPCTVN**Y** | WHYPCTVN**Y** | WHYPCTVN**Y** | 77.78% |
| YRYRFNSTG | Y**YHK**FNSTG | Y**YHK**FNSTG | Y**YHK**FNSTG | Y**YHK**FNSTG | | 66.67% | Y**YHK**FNS**S**G | Y**HH**RFNS**S**G | Y**YHK**FNS**S**G | Y**YNK**FNS**S**G | Y**YHK**FNS**S**G | 55.56% |
| FTLFKVRMF | **Y**TLFKVRMF | FT**S**FKVRMF | FTLFKVRMF | FTLFK**M**R**T**F | | 77.78% | **Y**TLFKVRM**Y** | **Y**TLFKVRM**Y** | **Y**TLFKVRM**Y** | **Y**TLFK**I**RM**Y** | **Y**TLFKVRM**Y** | 66.67% |
| VGGFEHRLS | VGG**L**EHR**FD** | VGGFEHR**FD** | VGGFEHR**FN** | VGGFEHR**FT** | | 66.67% | VGG**V**EHRL**E** | VGG**V**EHRL**E** | VGG**V**EHRL**E** | VGG**V**EHRL**Q** | VGG**V**EHRL**E** | 77.78% |
| YCFTPSPVV | YCFTPSPVV | YCFTPSPVV | YCFTPSPVV | YCFTPSPVV | | 100.00% | YCFTPSPVV | YCFTPSPVV | YCFTPSPVV | YCFTPSPVV | YCFTPSPVV | 100.00% |
| YPCTVNFTL | YPCTVN**Y**TL | YPCTVNFT**S** | YPCTVNFTL | YPCTVNFTL | | 88.89% | YPCT**I**N**Y**TL | YPCTVN**Y**TL | YPCTVN**Y**TL | YPCTVN**Y**TL | YPCTVN**Y**TL | 77.78% |
| FEHRLSAAC | **L**EHR**FD**AAC | FEHR**FD**AAC | FEHR**FN**AAC | FEHR**FT**AAC | | 66.67% | **V**EHRL**E**AAC | **V**EHRL**EV**AC | **V**EHRL**E**AAC | **V**EHRL**Q**AAC | **V**EHRL**EV**AC | 66.67% |
| IEDRDRSEQ | IEDRDRSEQ | **V**EDRDRSEQ | IEDRDRSEQ | IEDRDRSEQ | | 88.89% | **LD**DRDRSE**L** | **LD**DRDRSE**L** | **LD**DRDRSE**L** | **LD**DRDRSE**L** | **LD**DRDRSE**L** | 66.67% |
| YCWHYAPRS | YCWHY**P**PR**P** | YCWHYAPR**P** | YCWHYAPR**P** | YCWHYAPR**R** | | 77.78% | YCWHY**P**P**KP** | YCWHY**P**P**KP** | YCWHY**P**P**KP** | YCWHYAPR**P** | YCWHY**P**P**KP** | 66.67% |
| YGGNGRRGN | YGG**EK**R**ET**N | YGG**G**G**NPQ**N | YGG**E**G**SHKD** | YGG**G**G**NPH**N | | 44.44% | **I**GG**V**G**NNTL** | **I**GG**A**G**NNTL** | **I**GG**V**G**NNTL** | **V**G**N**N**TL**R**CP** | Absent | 0.00% |
| FRKHPEATY | FRKHP**G**ATY | FRKHPEATY | FRKHPEAT**C** | FRKHPEATY | | 88.89% | FRKHPEATY | FRKHPEATY | FRKHPEATY | FRKHPEATY | FRKHPEATY | 100.00% |
| YRLWHYPCT | YRLWHYPCT | YRLWHYPCT | YRLWHYPCT | YRLWHYPCT | | 100.00% | YRLWHYPCT | YRLWHYPCT | YRLWHYPCT | YRLWHYPCT | YRLWHYPCT | 100.00% |
| VNFTLFKVR | VN**Y**TLFKVR | VNFT**S**FKVR | VNFTLFKVR | VNFTLFK**M**R | | 88.89% | **I**N**Y**TLFKVR | VN**Y**TLFKVR | VN**Y**TLFKVR | VN**Y**TLFK**I**R | VN**Y**TLFKVR | 77.78% |
| IYRYRFNST | **F**Y**YHK**FNST | IY**YHK**FNST | **F**Y**YHK**FNST | **F**Y**YHK**FNST | | 55.56% | **F**Y**YHK**FNS**S** | **F**Y**HH**RFNS**S** | **F**Y**YHK**FNS**S** | **F**Y**YNK**FNS**S** | **F**Y**YHK**FNS**S** | 44.44% |
| IAGLIYRYR | IAGL**F**Y**YHK** | IAGLIY**YHK** | IAGL**F**Y**YHK** | IAGL**F**Y**YHK** | | 55.56% | IAGL**F**Y**YHK** | IAGL**F**Y**HH**R | **V**AGL**F**Y**YHK** | IAGL**F**Y**YNK** | **V**AGL**F**Y**YHK** | 44.44% |

**Note**: The amino acid residues which vary in other countries are shown in bold face while matching residues are shown in regular face. Minimum identity is identity percentage of least identical peptide sequence among various countries.
